# Acute and Subacute Oral Toxicity Evaluation of* Eriobotrya japonica* Leaf Triterpene Acids in ICR Mice

**DOI:** 10.1155/2017/4837839

**Published:** 2017-03-16

**Authors:** Feng Li, Yijia Li, Qingxian Li, Xianai Shi, Yanghao Guo

**Affiliations:** ^1^College of Chemistry, Fuzhou University, Fuzhou 350116, China; ^2^Institute of Pharmaceutical Biotechnology and Engineering, Fuzhou University, Fuzhou 350116, China

## Abstract

The interest focusing on* Eriobotrya japonica *leaf triterpene acid (ELTA) has increased recently because of its beneficial effects on health. However, there has been a lack of experimental data on its toxicity. The present study therefore was conducted to evaluate its toxicity in ICR mice. The results showed that ELTA produced neither mortality nor toxicity of the main organs in ICR male and female mice in both acute (0.30, 0.65, 1.39, and 3.00 g·kg^−1^ body weight) and subacute (150, 300, and 600 mg·kg^−1^ BW) 28-day toxicity studies. Because of lacking apparently adverse effects found in the hematology, clinical biochemistry, and histopathology evaluation, results of the present study together with the beneficial effects make ELTA as a promising dietary supplement and indicated that ELTA administered orally might have a large safety margin for human.

## 1. Introduction

**Figure 1:**
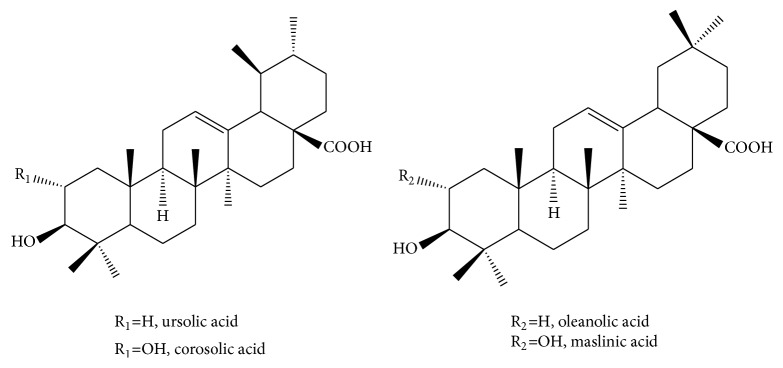
Chemical structures of the major triterpene acids of* Eriobotrya japonica* leaf.

*Eriobotrya japonica *leaf is included in the Chinese Pharmacopoeia [1] and widely used as a medicinal material in traditional treatment of a variety of chronic diseases in China and other East Asian countries [2]. Phytochemical investigations showed that its main components are essential oil, triterpenes, sesquiterpenes, flavonoids, tannins, and megastigmane glycosides [3]. Among them, triterpene acids are considered as the key pharmacological components. To date, more than twenty triterpene acids in* Eriobotrya japonica *leaf have been identified, including the major four triterpene acids, that is, ursolic acid, corosolic acid, oleanolic acid, and maslinic acid (Figure 1) [4, 5], which belong to ursane type and oleanane type.

Recently, there is increasing interest focusing on* Eriobotrya japonica *leaf triterpene acid (ELTA) because of its beneficial effects on health. ELTA have showed multiple pharmacological effects, including antitumor [6], antioxidative [7], hypoglycemic, and hypolipidemic effects [8]. However, to our knowledge, the safety of ELTA has not been assessed yet in spite of its beneficial effects on health. Although Sánchez-González et al. [9] have made a preliminary safe evaluation of maslinic acid from olive, the experimental data about the toxicity of ELTA are still insufficient. Even with a long history of use, a medicinal plant could probably also induce unexpected toxicity during its use if it was not systematically safety-evaluated. In traditional Chinese medicine,* Eriobotrya japonica *leaf preparations are mostly water-soluble preparations, that is, Chuanbei Loquat Cream. But for non-water-soluble ELTA, we know little about its safety. Therefore, we assessed the possible toxic effects of ELTA on male and female ICR mice through single oral administrations of acute study and 28 consecutively daily oral administrations of subacute study in the present study.

## 2. Materials and Methods

### 2.1. Materials

*Eriobotrya japonica *leaf medicinal material was purchased from Hui Chun Pharmaceutical Co., Ltd. (Fuzhou, China). 2-hydroxypropyl-*β*-cyclodextrin (HPCD), sodium carboxymethyl cellulose (CMC-Na), and standards of triterpene acid were purchased from Aladdin Co., Ltd. (Shanghai, China). Methanol (HPLC grade) and NH_4_HCO_3_ (AR grade) was purchased from Xilong Chemical Co., Ltd. (Guangzhou, China). Water was obtained from a Kertone Lab mini water purification system (Kertone Co., Ltd., Changsha, China). Unless otherwise noted, other reagents were of AR grade.

### 2.2. Preparation of the ELTA

The* Eriobotrya japonica* leaves were dried at 45°C overnight using an oven with air circulation. The dried leaves were ground into a fine powder. ELTA was prepared according to the following steps. The powder was extracted with 95% ethanol three times at 60°C for 2 h. The extracting solution was collected using a Buchner funnel. After being decolored by adding 1.5% (m/v) activated carbon powder at pH 10 for 30 min and being filtered, the extracting solution changed from green to brown, and then the solvent was diluted with water to 30% ethanol. The diluted extracting solution was chromatographed with HZ816 macroreticular resin using a gradient eluant of H_2_O-ethanol (H_2_O, 10%, 30%, 60%, and 90% ethanol). The 30% and 60% ethanol eluant fractions were collected and neutralized to pH 7.0 and then centrifuged at 6000 ×g for 10 min to obtain the precipitate of total triterpene acids. The precipitate was dried at 60°C and obtained ELTA.

The contents of major four triterpene acids in ELTA were analyzed by HPLC method. The analysis was conducted using a LC-20A (Shimadzu, Japan) equipped with a SDLC18BB5051 column (5 *μ*m, 250 × 4.6 mm). The mobile phase consisted of methanol and 5 mmol/L NH_4_HCO_3_ (80/20,* v*/*v*), and the mobile phase flow rate was 1.0 mL/min. The contents of major four triterpene acids in ELTA were calculated according to the peak area of standard triterpene acid, respectively. The content are listed as follows: maslinic acid: 161.85 mg/g ELTA, corosolic acid: 300.30 mg/g ELTA, oleanolic acid: 91.65 mg/g ELTA, and ursolic acid: 391.95 mg/g ELTA, and the content of total triterpene acids in ELTA was more than 94%.

### 2.3. Animals and Treatment

All animals used in the present study were handled in accordance with the National Institutes of Health (NIH) Guide for the Care and Use of Laboratory Animals. The experimental protocols were developed on the basis of above regulations and approved by Animal Ethics Committee of the Fuzhou University. All possible efforts were made to minimize the animals' suffering and to reduce the number of animals used.

Young adult ICR mice (6 weeks old, weighing 20–22 g) were from Shanghai Slac Laboratory Animal Center (number of animal license: SCXK HU 2012-0002). Following one week of acclimatization period, the mice were randomly distributed to the control and the treated groups and housed in standard environmental conditions. The temperature of animal room was 22–25°C and the relative humidity arranged from 40–60%. The animal room was set in a 12 h light/dark cycle. A standard diet and water were provided to animals ad libitum.

### 2.4. Acute Toxicity Evaluation

In this work, Horn's method was used to assess the acute toxicity of ELTA. After the acclimatization period, 20 male and 20 female ICR mice were randomly divided into 4 groups of 5 males or 5 females, respectively. ELTA was dissolved in a solution of 30% 2-hydroxypropyl-*β*-cyclodextrin (HPCD) and 0.5% sodium carboxymethyl cellulose (CMC-Na) and administered by oral gavage once daily at dosages of 0.30, 0.65, 1.39, and 3.00 g·kg^−1^ BW, respectively (with 0.1 mL per 10 g body weight). As for the dosage of 3.00 g·kg^−1^ BW, twice daily (with a 4 h interval) administration was adopted due to its concentration limit. The control group only received the vehicle solvent. Vehicle or ELTA was administered only once. The relevant clinical symptoms were closely monitored during the first six hours. The animals were then observed for their toxic symptoms, behavior changes, and mortality at least once daily for 14 days. Body weights were measured before or at the end of this experiment. At the end of this period, the remaining mice were weighed and euthanized by* i.p.* injection of sodium pentobarbital (50 mg·kg^−1^ BW), and visceral organs were excised for a gross pathological examination.

### 2.5. Subacute Toxicity Evaluation

The subchronic toxicity evaluation was conducted according to the Technical Guidelines of Traditional Chinese Medicine and Natural Medicine Chronic Toxicity Studies GPT3-1 (in Chinese) (China Food and Drug Administration, 2005). Twenty male and twenty female ICR mice were divided into four groups, respectively (5 animals in each group), one control group and three treated groups. The doses of treated groups were determined according to the results of the acute oral toxicity test. The control group was administered with vehicle by oral gavage once daily, and treated groups were administered with ELTA at three doses (150, 300, and 600 mg·kg^−1^ bw), respectively (with 0.1 mL per 10 g body weight). Vehicle or ELTA was administered at the same time every day for 28 days and each mouse was observed daily for clinical symptoms such as piloerection, abnormal mucosal secretion, and changes in respiratory and locomotor systems,. The animals were weighed every 3 days, and food and water consumption were recorded every 2 days.

#### 2.5.1. Hematological and Serum Biochemical Examination

On day 29, mice were anesthetized by* i.p.* injection of sodium pentobarbital (50 mg·kg^−1^ BW), and the blood samples were collected in sterile tubes for hematology and clinical biochemistry assay. Blood samples collected in tubes containing anticoagulant were used for hematology analysis. Red blood cell count (RBC), white blood cell count (WBC), hemoglobin (HGB), hematocrit (HCT), mean corpuscular volume (MCV), mean corpuscular hemoglobin (MCH), mean corpuscular hemoglobin concentration (MCHC), platelets (PLT), lymphocytes (LYM), and monocytes (MONO) were analyzed using automatic hematology analysis system. Blood samples without anticoagulant were used for serum biochemistry analysis, the samples were placed at room temperature for 1 h and then centrifuged at 1500 ×g for 10 min to obtain serum. The serum was used for analyzing the following parameters: aspartate aminotransferase (AST), alanine aminotransferase (ALT), creatinine (CRE), blood glucose (GLU), triglyceride (TG), total cholesterol (TCHO), sodium, potassium, and chloride.

#### 2.5.2. Organ Weight and Histopathology Assay

Gross necropsy was performed for each animal, including careful examination of external surfaces, orifices, thoracic and abdominal cavities, and their contents. Liver, kidneys, heart, lung, spleen, testicles, and ovaries were weighed individually and the relative organ weights were calculated according to the following formula: relative organ weight (%) = weight of organ (g)/body weight (g) × 100. For histopathological examinations, organs were fixed in 10% formamide before being embedded in paraffin. After routine processing, five micrometers of paraffin sections were prepared and stained with hematoxylin and eosin before microscopic examination.

### 2.6. Statistical Analysis

The data are expressed as mean ± SD. Statistical analysis was conducted by one-way ANOVA followed by Tukey's test to evaluate significant differences between the control and the treated groups. *p* < 0.05 was considered statistically significant.

## 3. Results

### 3.1. Acute Oral Toxicity Evaluation of ELTA

In this acute toxicity study, no mortality was observed for the following 14 days after the administrations of all single doses of ELTA, and no treatment-related clinical symptoms of toxicity were observed for either a short period (48 h) or a long period (14 days) at each dose of 0.30, 0.65, and 1.39 g ELTA·kg^−1^ BW (Table 1). When the dose came to 3.00 g·kg^−1^ BW, mild piloerection symptom was monitored in 1/5 male and 2/5 female mice, but the symptom disappeared within 48 h after the drug administration. In addition, all the administrations of single dose did not cause body weight loss (Table 1), and there was not any gross pathological change of internal organs observed in necropsy examination.

### 3.2. General Clinical Symptom and Mortality of Mice in Subacute Toxicity Study

In the repeated dose 28-day oral toxicity study, no obvious clinical symptoms were observed during the experimental period at any of the three doses. No mortality was observed during the 28 days, likewise. No differences in general behavior were observed between the control and the ELTA treated groups.

### 3.3. Body Weight of the Mice in Subacute Study

**Figure 2:**
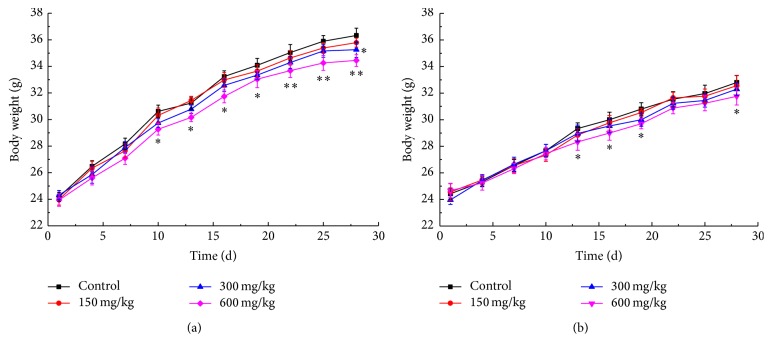
Effect of ELTA on body weight of male (a) and female (b) ICR mice during 28 days of repeated dose toxicity study. Data are expressed as means ± SD (*n* = 5) and analyzed by one-way ANOVA (^*∗*^*p* < 0.05 versus control; ^*∗∗*^*p* < 0.01 versus control).

The mean body weight changes of male and female mice are shown in Figure 2. For male mice, although ELTA appeared to reduce their body weight at doses of 150 and 300 mg·kg^−1^ BW, no statistically significant differences were recorded. However, ELTA significantly reduced body weight from the 11th day to the last day (*p* < 0.05) at 600 mg·kg^−1^ BW (Figure 2(a)). As to female mice, the results were similar to those of male mice. There were no significant differences observed between the control and the treated groups at the doses of 150 and 300 mg·kg^−1^ BW, although the treated animals appeared to have an obvious smaller body weight from the 14th day to the last. But at the dose of 600 mg·kg^−1^ BW, the treated animals' body weights were significantly reduced (*p* < 0.05) (Figure 2(b)).

### 3.4. Necropsy Macroscopic Observation and Relative Organ Weights

In the necropsy examination, no treatment-related pathological changes of internal organs were observed in male and female mice administrated with ELTA.

As to relative organ weights, there were also no significant differences observed (*p* > 0.05) (Table 2).

### 3.5. Histopathological Examination

**Figure 3:**
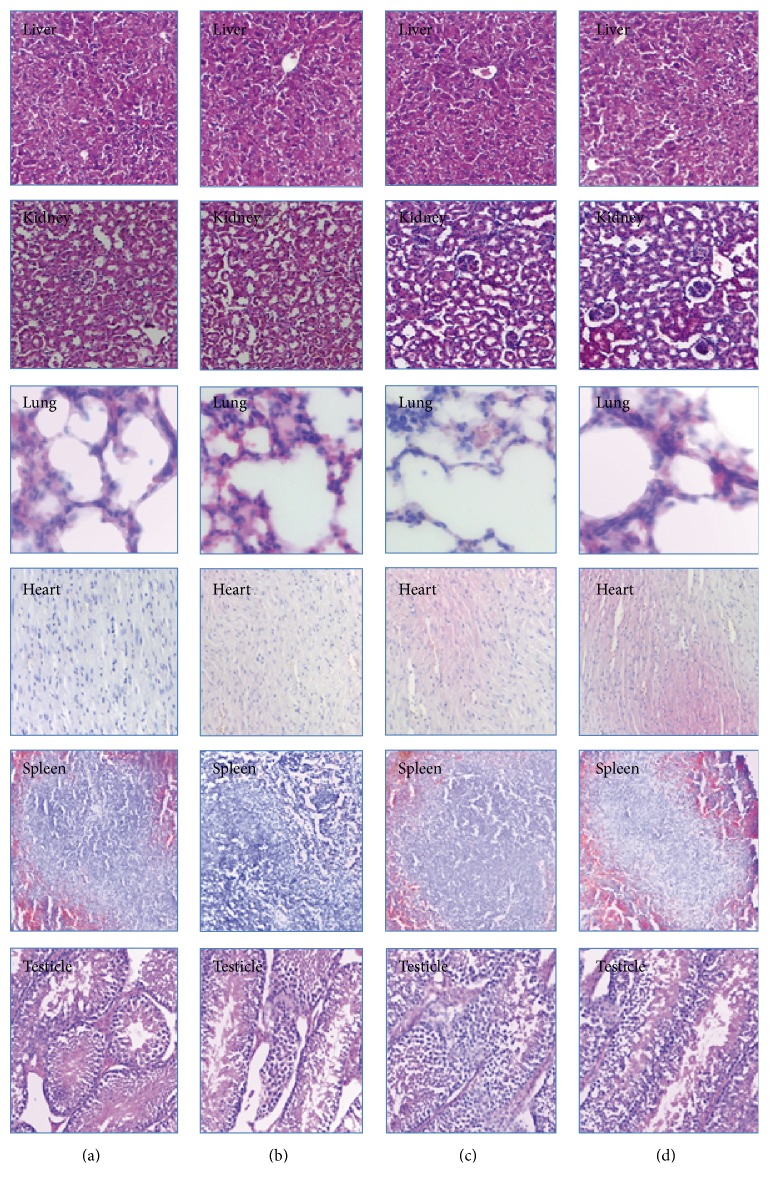
Effects of ELTA on histological examinations of the main organs in mice during 28 days of repeated dose toxicity study. Representative photomicrographs from liver, kidney, heart, spleen, and testicle sections stained with hematoxylin and eosin (×200), respective groups: (a) male control group, (b) male treated group (600 mg·kg^−1^ BW ELTA), (c) female control group, and (d) female treated group (600 mg·kg^−1^ BW).

A histological examination of the main organs was performed at the end of subacute study period.  Figure 3 shows the representative photomicrographs of organs from the mice of control and ELTA treated groups. H-E staining of liver showed normal hepatic architecture, hepatocytes, and perilobular vein. Histological assay of the kidney from ELTA treated groups showed a normal renal architecture with a normal appearance of glomerulus and tubules. Heart sections of ELTA treated groups presented a normal myocardial architecture. Assessment of the lung sections displayed normal appearance with normal bronchiole and alveolus. The spleen sections displayed normal architecture with lymphoid follicles and sinuses. The testicles sections showed a normal appearance of sperm and seminiferous tubule in the ELTA treated groups. In short, the histological assay showed that ELTA produced no significant pathological organ lesions during the subacute study period.

### 3.6. Serum Biochemical Profile Analysis

In general, the 28-day repeated administration of ELTA did not produce treatment-related biologically significant adverse effects on serum biochemistry parameters in male and female mice (Table 3). However, as compared to the control group, some statistical differences were observed in some treated groups. For male mice, a statistical increase in blood sodium at doses of 150 and 600 mg·kg^−1^ BW and a statistical increase in blood potassium at the dose of 300 mg·kg^−1^ BW were observed. Yet there were no coincident histological changes of kidney observed in the present study (Figure 3). As compared to the control group, administration of ELTA at 600 mg·kg^−1^ BW produced statistical decreases in alanine amino transferase and aspartate aminotransferase. Because increase in the activities of such aminotransferases represents liver damage, this could not be regarded as an evidence that ELTA has toxicity on liver.

For female mice, a statistical increase in blood potassium at 150 mg·kg^−1^ BW and a statistical increase in blood sodium at 300 mg·kg^−1^ were observed, but these results were not reflected in concurrent histopathological changes in kidney (Figure 3). As compared to the control group, administration of ELTA at 600 mg·kg^−1^ BW produced statistical decreases in triglyceride, total cholesterol, and aspartate aminotransferase. As there were no coincident histopathological changes observed in liver and in kidney, these results could not be considered any of toxicity.

Taken together, these statistical differences of several serum chemical parameters were not dose-related or reflected by other coincident data, and these changes could be considered within normal experimental range and as incidental.

### 3.7. Hematological Profile Analysis

The levels of hematological parameters in male and female mice are shown in Table 4. No treatment-related differences in hematology were observed. However, some statistical changes were observed in the treated groups. For male mice, administration of ELTA at 150 mg·kg^−1^ BW produced statistical changes in white blood cells (increased) and mean corpuscular volume (decreased). It produced a statistical difference in mean corpuscular hemoglobin concentration (decreased) at the dose 300 mg·kg^−1^ BW and produced statistical differences in mean corpuscular volume (decreased) and mean corpuscular hemoglobin (decreased) at the dose of 600 mg·kg^−1^ BW. For female mice, administration of ELTA produced a statistical change in mean corpuscular hemoglobin concentration (increased) at the dose of 150 mg·kg^−1^ BW and produced a significant change in mean corpuscular volume (decreased) at the dose of 600 mg·kg^−1^ BW. As such statistical differences were not dose-related, they were not considered as treatment-related changes.

## 4. Discussion

The four major triterpene acids of* Eriobotrya japonica *leaf have received more and more attention recently. The related pharmacological studies were focused on antitumor [10–13], antioxidation [14, 15], antidiabetes [16–18], anti-inflammation [19–21], and improving metabolic syndrome [21, 22]. In Chinese folk and traditional Chinese medicine, though* Eriobotrya japonica *leaf is widely used in traditional treatment of various diseases such as hyperlipidemia, hyperglycemia, and inflammation, there is little research data on the safety of* Eriobotrya japonica* leaf extracts. In the present study, we evaluated the acute toxicity of ELTA on health using Horn's method (at doses of 0.30, 0.65, 1.39, and 3.00 g·kg^−1^ BW) and evaluated the subacute toxic effects using repeated dose 28-day oral toxicity study (at doses of 150, 300, and 600 mg·kg^−1^ BW).

The acute toxicity was researched in order to determine whether administration of single high dose of ELTA brought any adverse effect on tested animals during the immediate two weeks after the oral administration. Mild piloerection symptom was observed in several mice at the highest dose of 3.00 g·kg^−1^ BW (lasting no more than 48 h), suggesting that ELTA may cause some short-term stress to automatic nervous system at high doses. Except this, ELTA did not produce any mortality, adverse clinical symptoms, abnormal changes in behavior, macroscopic findings, or food consumption during the acute toxicity testing. No mortality was observed at the highest dose, so LD_50_ for ELTA was higher than 3.00 g·kg^−1^ BW for both the male and female ICR mice. These results suggested that ELTA is practically nontoxic under these conditions. According to related criteria of acute classification [23], ELTA is not classified as toxicant.

The doses employed in subacute toxicity study were selected according to LD_50_ calculated in the acute toxicity study. The ICR mice were administered by oral gavage daily with ELTA at three doses (1/20 of LD_50_, 1/10 of LD_50_, and 1/5 of LD_50_), respectively. Daily oral administration of ELTA did not produce any symptom of toxicity, or mortality in the treated mice. But the body weights of the treated mice were significantly decreased at dose of 600 mg·kg^−1^ BW when compared to the control mice, and this result was in agreement with previous related studies [24, 25] and confirmed the antiobese effect of ELTA. At the end of the subacute toxicity experiment, all groups looked uniformly healthy. These results further showed that ELTA should be considered to be nontoxic in mice at these doses employed in the present study.

Relative organ weight is an important index to determine whether the organ has been exposed to injury [26]. In this work, relative weights of liver, kidneys, heart, lung, spleen, thymus, testicles, or ovaries of the treated groups were not statistically different from the control group. No treatment-related gross macroscopic findings were observed in the necropsy examination. Likewise, there was no toxic effect in the histopathological evaluation.

Serum biochemistry was analyzed in order to assess the overall health status and the alterations in metabolic processes. Serum biochemical analysis showed that ELTA could decrease AST, ALT, TG, and TCHO at the high dose of 600 mg·kg^−1^ BW, and these results were consistent with the previous related studies [27–30], further confirming liver-protective effect and antihyperlipidemia effects of triterpene acids. Though there were several minor changes of ion levels such as sodium and potassium observed, these results were not reflected in coincident histopathological changes in kidneys. Serum concentration of creatinine regarded as a marker of kidney integrity showed the absence of adverse effects of ELTA in renal function. Plus they were not dose-related, so they could be considered as incidental.

Blood parameter analysis is very important in evaluation of the hematological lesions [31]. Changes in hematological parameters can sometimes reflect lesions of the hematopoietic system [32], inflammatory reactions [33]. Though statistical changes were observed in several blood parameters of the treated groups (Table 4), such differences were not dose-related, so they were considered as incidental and not treatment-related.

## 5. Conclusion

In acute (0.30, 0.65, 1.39, and 3.00 g·kg^−1^ BW) toxicity study, except that mild piloerection symptom was observed at the highest dose, ELTA did not produce any mortality, adverse clinical symptoms. In subacute (150, 300, and 600 mg·kg^−1^ BW) toxicity studies, ELTA did not produce any mortality and toxicity of the main organs in ICR male and female mice. Because of lacking apparently adverse effects found in the hematology, clinical biochemistry, and histopathology evaluation, results of the present study indicate that ELTA administered orally might have a large safety margin for human. Due to absence of information concerning mutagenicity and genotoxicity, there are still more studies needed to be conducted in order to utilize ELTA as a beneficial product for health.

## Figures and Tables

**Table 1:** Acute oral toxicity study of ELTA in ICR mice. All data are expressed as means ± SD (*n* = 5 in each group), *p* > 0.05 for all ELTA treated groups compared with the control group. ^a^Mild piloerection symptom was observed in 1/5 mice and disappeared within 48 h after drug administration. ^b^Mild piloerection symptom was observed in 2/5 mice and disappeared within 48 h after drug administration.

Sex	ELTA dose (g·kg^−1^ BW)	Body weight (g)		Dead/total	Symptoms
		1st day	14th day		
Male	0.30	23.84 ± 0.87	31.25 ± 1.18	0/5	None
	0.65	24.26 ± 1.33	30.65 ± 1.67	0/5	None
	1.39	24.16 ± 1.04	30.24 ± 1.02	0/5	None
	3.00	23.93 ± 0.75	29.64 ± 1.52	0/5	Piloerection^a^

Female	0.30	22.94 ± 0.84	28.51 ± 1.28	0/5	None
	0.65	22.31 ± 1.12	28.34 ± 0.75	0/5	None
	1.39	22.69 ± 0.73	28.02 ± 0.84	0/5	None
	3.00	22.54 ± 0.81	27.73 ± 1.06	0/5	Piloerection^b^

**Table 2:** Relative organ weights (% body weight) of control and ELTA treated mice by gavage consecutively for 28 days. Data are expressed as means ± SD (*n* = 5) and analyzed by one-way ANOVA, *p* > 0.05 for all ELTA treated groups compared with the control group. Relative organ weight (%) = (organ weight/28-day body weight) × 100.

Sex	Organ (% body weight)	Control	ELTA dose		
			150 mg·kg^−1^ BW	300 mg·kg^−1^ BW	600 mg·kg^−1^ BW
Male	Liver	4.94 ± 0.21	5.03 ± 0.17	5.11 ± 0.18	5.08 ± 0.17
	Kidneys	1.21 ± 0.02	1.26 ± 0.03	1.18 ± 0.02	1.24 ± 0.04
	Heart	0.48 ± 0.03	0.50 ± 0.04	0.51 ± 0.02	0.55 ± 0.02
	Lung	0.65 ± 0.04	0.63 ± 0.05	0.64 ± 0.02	0.59 ± 0.05
	Spleen	0.35 ± 0.01	0.33 ± 0.03	0.31 ± 0.04	0.30 ± 0.05
	Testicles	0.97 ± 0.07	1.08 ± 0.04	1.15 ± 0.10	0.98 ± 0.03
	Thymus	0.18 ± 0.03	0.19 ± 0.01	0.17 ± 0.03	0.17 ± 0.6

Female	Liver	4.83 ± 0.18	4.94 ± 0.27	5.15 ± 0.28	5.11 ± 0.25
	Kidneys	1.15 ± 0.06	1.24 ± 0.12	1.22 ± 0.15	1.15 ± 0.05
	Heart	0.51 ± 0.07	0.49 ± 0.03	0.52 ± 0.04	0.51 ± 0.04
	Lung	0.63 ± 0.07	0.71 ± 0.11	0.73 ± 0.06	0.72 ± 0.05
	Spleen	0.38 ± 0.05	0.34 ± 0.06	0.31 ± 0.02	0.29 ± 0.03
	Ovaries	0.10 ± 0.01	0.10 ± 0.01	0.09 ± 0.02	0.09 ± 0.01
	Thymus	0.24 ± 0.03	0.21 ± 0.02	0.19 ± 0.03	0.18 ± 0.05

**Table 3:** Serum biochemistry parameters of control and ELTA treated mice by gavage consecutively for 28 days. Data are expressed as means ± SD (*n* = 5) and analyzed by one-way ANOVA (^*∗*^*p* < 0.05 versus control). GLU = blood glucose; TG = triglyceride; TCHO = total cholesterol; AST = aspartate aminotransferase; ALT = alanine aminotransferase; CRE = creatinine.

Sex	Biochemistry parameters	Control	ELTA dose		
			150 mg·kg^−1^ BW	300 mg·kg^−1^ BW	600 mg·kg^−1^ BW
Male	GLU (mmol/L)	10.28 ± 1.34	10.04 ± 0.65	9.84 ± 1.18	9.67 ± 0.84
	TG (mmol/L)	0.76 ± 0.10	0.69 ± 0.07	0.71 ± 0.06	0.65 ± 0.12
	TCHO (mmol/L)	3.58 ± 0.25	3.10 ± 0.17	3.15 ± 0.22	3.21 ± 0.15
	AST (U/L)	115 ± 11	113 ± 18	97 ± 13	92 ± 12^*∗*^
	ALT (U/L)	42 ± 6	40 ± 5	36 ± 4	32 ± 4^*∗*^
	CRE (*μ*mol/L)	18.7 ± 2.1	15.4 ± 1.7	16.3 ± 2.5	16.8 ± 2.6
	Sodium (mmol/L)	146.4 ± 3.2	153.7 ± 2.3^*∗*^	146.6 ± 2.5	152.3 ± 1.9^*∗*^
	Potassium (mmol/L)	5.52 ± 0.41	6.34 ± 0.69	6.52 ± 0.32^*∗*^	5.97 ± 0.41
	Chloride (mmol/L)	104.6 ± 4.5	98.6 ± 6.3	101.1 ± 3.2	97.6 ± 5.1

Female	GLU (mmol/L)	10.78 ± 0.74	10.35 ± 0.45	10.23 ± 0.98	9.58 ± 0.56
	TG (mmol/L)	0.84 ± 0.08	0.72 ± 0.11	0.67 ± 0.12	0.63 ± 0.16^*∗*^
	TCHO (mmol/L)	3.81 ± 0.22	3.34 ± 0.23	3.45 ± 0.27	3.12 ± 0.22^*∗*^
	AST (U/L)	112 ± 15	104 ± 13	108 ± 17	95 ± 8^*∗*^
	ALT (U/L)	27 ± 5	31 ± 5	24 ± 6	25 ± 3
	CRE (*μ*mol/L)	16.3 ± 2.4	14.6 ± 1.4	15.4 ± 2.0	15.7 ± 1.9
	Sodium (mmol/L)	147.5 ± 1.2	147.2 ± 1.1	152.6 ± 0.8^*∗*^	148.4 ± 1.3
	Potassium (mmol/L)	6.02 ± 0.31	7.24 ± 0.45^*∗*^	6.33 ± 0.25	6.71 ± 0.21
	Chloride (mmol/L)	103.31 ± 3.52	105.77 ± 4.34	105.24 ± 2.64	104.38 ± 2.14

**Table 4:** Hematological parameters of control and ELTA treated mice by gavage consecutively for 28 days. Data are expressed as means ± SD (*n* = 5) and analyzed by one-way ANOVA (^*∗*^*p* < 0.05 versus control). RBC = red blood cells; WBC = white blood cells; HGB = hemoglobin; HCT = hematocrit; MCV = mean corpuscular volume; MCH = mean corpuscular hemoglobin; MCHC = mean corpuscular hemoglobin concentration; PLT = platelets; LYM = lymphocytes; MONO = monocytes.

Sex	Hematological parameters	Control	ELTA dose		
			150 mg·kg^−1^ BW	300 mg·kg^−1^ BW	600 mg·kg^−1^ BW
Male	RBC (10^12^/L)	7.58 ± 1.43	7.95 ± 0.84	8.14 ± 1.51	7.83 ± 0.94
	WBC (10^9^/L)	7.36 ± 0.93	8.96 ± 0.53^*∗*^	6.71 ± 1.21	6.96 ± 1.33
	HGB (g/L)	142 ± 15	127 ± 8	137 ± 19	135 ± 13
	HCT (%)	47.9 ± 5.4	51.3 ± 3.2	46.5 ± 3.9	48.7 ± 4.1
	MCV (fL)	51.6 ± 1.8	43.8 ± 1.3^*∗*^	52.3 ± 1.7	43.5 ± 1.6^*∗*^
	MCH (pg)	15.8 ± 0.6	15.3 ± 0.3	15.5 ± 0.7	13.8 ± 0.4^*∗*^
	MCHC (g/dL)	29.7 ± 1.5	30.4 ± 0.7	33.5 ± 0.6^*∗*^	31.4 ± 0.7
	PLT (10^9^/L)	657 ± 57	586 ± 94	614 ± 73	627 ± 61
	LYM (%)	76.2 ± 3.3	80.0 ± 4.1	78.7 ± 2.5	81.3 ± 2.8
	MONO (%)	2.64 ± 0.57	1.86 ± 0.62	2.48 ± 0.47	2.34 ± 0.35

Female	RBC (10^12^/L)	7.38 ± 1.17	7.27 ± 0.76	7.64 ± 1.35	6.83 ± 1.24
	WBC (10^9^/L)	7.41 ± 1.23	7.26 ± 0.93	7.17 ± 0.70	7.57 ± 0.32
	HGB (g/L)	130 ± 13	119 ± 10	122 ± 9	115 ± 14
	HCT (%)	45.4 ± 3.1	46.9 ± 2.8	47.8 ± 4.0	43.2 ± 4.3
	MCV (fL)	54.4 ± 1.2	55.3 ± 1.0	52.9 ± 0.9	44.2 ± 1.3^*∗*^
	MCH (pg)	16.2 ± 0.7	17.3 ± 0.5	16.8 ± 0.6	15.4 ± 0.7
	MCHC (g/dL)	30.6 ± 1.6	35.2 ± 0.7^*∗*^	29.7 ± 1.6	33.2 ± 0.8
	PLT (10^9^/L)	637 ± 53	657 ± 49	594 ± 37	627 ± 45
	LYM (%)	77.9 ± 3.7	78.3 ± 5.6	73.5 ± 6.1	72.4 ± 3.5
	MONO (%)	2.56 ± 0.38	2.86 ± 0.31	2.38 ± 0.17	2.75 ± 0.28
